# Ethical Governance of Artificial Intelligence in Cardiovascular Disease Management: A Health Policy Perspective

**DOI:** 10.7759/cureus.103995

**Published:** 2026-02-20

**Authors:** CFC Ogbuefi, OL Ezika, JO Egbunike, KE Ogbuefi

**Affiliations:** 1 Department of Family Medicine, Federal University Teaching Hospital, Owerri, NGA; 2 Department of Family Medicine, Federal Medical Center Jabi, Abuja, NGA; 3 Department of Family Medicine, Federal Medical Center, Umuahia, NGA

**Keywords:** artificial intelligence, cardiovascular disease, health equity, health ethics, health policy

## Abstract

Artificial intelligence (AI) is increasingly integrated into cardiovascular disease (CVD) prevention, diagnosis, and risk stratification, offering opportunities to improve early detection and population health outcomes. However, rapid adoption of AI technologies has outpaced the development of ethical, regulatory, and equity-centered governance frameworks, raising concerns about algorithmic bias, transparency, interoperability, and public trust. This editorial examines challenges in the governance of AI for cardiovascular care in the United States and situates them within global digital health policy discussions. While current federal oversight primarily focuses on clinical safety, this editorial advances a policy-oriented framework, formulated by the authors as the Artificial Intelligence Driven Cardiovascular Health Equity and Data Integration Act (AI-CVD Equity Act). This proposed act builds upon the existing Food and Drug Administration (FDA) and Department of Health and Human Services (HHS) initiatives by introducing mandatory, standardized demographic bias audits and regionally coordinated community oversight with critical layers of governance currently absent from federal frameworks to support equitable, transparent, and accountable AI deployment. Strengthening governance, workforce capacity, and community engagement is essential to ensure that AI-driven innovation reduces, rather than exacerbates, cardiovascular health disparities.

## Editorial

Introduction

Cardiovascular disease (CVD) remains the leading cause of morbidity and mortality worldwide and a major driver of preventable healthcare costs in the United States [1]. Despite advances in pharmacologic therapies, interventional cardiology, and public health prevention strategies, substantial disparities persist across racial, socioeconomic, and geographic populations [2]. These inequities reflect structural determinants of health, fragmented preventive care, and limitations in traditional cardiovascular risk assessment tools. While these disparities are well-documented, a critical policy gap remains, which includes the lack of a standardized governance framework to prevent AI from scaling these historical biases. This editorial addresses this void by proposing a regionally coordinated legislative model, which is the Artificial Intelligence Driven Cardiovascular Health Equity and Data Integration Act (AI-CVD Equity Act), designed to bridge the gap between high-performance technology and equitable clinical outcomes.

Artificial intelligence (AI), including machine learning and deep learning approaches, has emerged as a powerful tool for analyzing large-scale clinical and population health data. AI-enabled models can integrate electronic health records (EHRs), electrocardiograms, imaging data, laboratory values, and wearable device outputs to generate individualized risk predictions and detect subclinical disease earlier than conventional methods [3,4]. In cardiovascular medicine, AI applications have demonstrated promising performance in identifying atrial fibrillation, ventricular dysfunction, and structural heart disease prior to clinical presentation [5-7]. While this technological potential offers a techno-optimistic path toward precision cardiology, its actual clinical utility and equitable reach are fundamentally dependent on the regulatory guardrails that govern its deployment.

However, the accelerated deployment of AI in healthcare has occurred in the absence of comprehensive governance mechanisms to ensure ethical use, transparency, and equity. Without deliberate safeguards, AI systems risk reinforcing historical biases embedded in healthcare data and widening existing cardiovascular disparities [8]. This editorial discusses key governance challenges and proposes a policy framework to guide equitable AI integration into CVD prevention.

Current challenges in AI governance for CVD

Current oversight of AI in US healthcare primarily emphasizes safety and efficacy through the Food and Drug Administration’s framework for AI and machine learning software as a medical device [9]. While this approach addresses clinical performance and post‑market monitoring, it does not require standardized assessments of algorithmic bias, demographic performance reporting, or mechanisms for community accountability.

Interoperability challenges further limit the effectiveness of AI-enabled CVD prevention. Fragmented EHR systems, heterogeneous state-level privacy regulations, and limited public health informatics capacity restrict data integration and the development of generalizable AI models. Public health agencies continue to face barriers in receiving and using electronic clinical data for surveillance and prevention activities [10].

These governance gaps directly impede clinical workflows. For instance, the lack of standardized performance reporting means a clinician integrating an AI-enabled risk score into a preventive screening workflow has no federal assurance that the tool maintains accuracy across diverse patient zip codes. While the FDA holds the authority to mandate such demographic reporting at the federal level, the fragmentation of data, which is often locked in EHR silos due to varying state-level privacy laws, requires institutional and state-level policy shifts to achieve true interoperability. Distinguishing these roles is vital as the federal authorities must set the "what" of demographic auditing, while institutional leaders must resolve the "how" of data integration to support real-time cardiovascular surveillance.

Additionally, gaps in workforce readiness and ethical oversight persist. Many clinicians and public health professionals lack formal training in AI literacy, bias evaluation, and interpretation of algorithmic outputs [11]. These deficiencies constrain the ability of health systems to implement AI responsibly and undermine public confidence in AI-driven care.

Ethical and equity considerations

Ethical integration of AI in cardiovascular care requires attention to fairness, transparency, accountability, and respect for patient autonomy. Evidence indicates that AI systems trained on historically biased datasets may systematically underperform for racial and ethnic minority populations, women, and socioeconomically disadvantaged groups [8,12]. The necessity for such governance is underscored by documented instances of algorithmic bias in cardiovascular care. A landmark 2019 study revealed that a widely utilized clinical algorithm systematically underestimated the health needs of Black patients by using healthcare spending as a proxy for illness severity. Because historical socioeconomic barriers led to lower spending for Black populations despite higher disease burdens, the AI incorrectly flagged them as healthier, potentially delaying critical interventions. Furthermore, traditional cardiovascular risk scores have long faced criticism for being developed on predominantly Caucasian cohorts, leading to significant risk misclassification in underrepresented groups. These cases demonstrate that without the mandatory bias audits and community oversight proposed in the AI-CVD Equity Act, AI integration risks scaling these historical inequities rather than resolving them [8]. Without corrective measures, such disparities risk being amplified at scale.

Global guidance, including recommendations from the World Health Organization, emphasizes the importance of human‑centered AI, transparency, and equity in digital health innovation [13]. These principles underscore the need for continuous performance auditing, explainability of AI outputs, and human‑in‑the‑loop clinical decision‑making. Translating these global principles into practical safeguards within US health systems requires institutionalizing ethical oversight at the point of care. Specifically, clinical departments should implement algorithmic impact assessments before adopting new cardiovascular AI tools, ensuring that the local patient demographics match the tool's training data. Furthermore, health systems must establish multidisciplinary AI ethics committees composed of cardiologists, data scientists, and community patient advocates to provide human-in-the-loop surveillance of AI outputs. By shifting from theoretical alignment to these concrete institutional protocols, US providers can ensure that AI serves as a reliable partner in reducing cardiovascular disparities rather than a passive observer of them. 

Policy and legislative recommendations

To address the existing governance gaps, this editorial proposes the AI‑CVD Equity Act. This framework emphasizes interoperable data infrastructure, mandatory demographic performance audits, workforce development, and structured community engagement to support equitable AI deployment.

Key elements of the proposed AI‑CVD Equity Act are summarized in Table 1.

**Table 1:** Key Components of the AI-CVD Equity Act AI, artificial intelligence; CVD, cardiovascular disease; AI-CVD Equity Act, Artificial Intelligence–Driven Cardiovascular Health Equity and Data Integration Act.

Component	Description	Expected Public Health Impact
National Cardiovascular AI Data Infrastructure	Federally coordinated, interoperable repository linking de-identified cardiovascular data from health systems and public health agencies	Improves generalizability of AI models and supports equitable early detection
Mandatory Algorithmic Bias Audits	Required demographic performance evaluation before and after AI deployment	Reduces risk of reinforcing racial, socioeconomic, and geographic disparities
Ethical and Governance Oversight	Independent ethics review, human-in-the-loop oversight, and public reporting	Enhances transparency, accountability, and public trust
Workforce Development and Training	Federally supported AI literacy and ethics training for clinicians and public health professionals	Strengthens responsible implementation and interpretation
Community Engagement Structures	Regional AI equity committees including community representatives	Promotes culturally responsive and trustworthy AI deployment

While the AI-CVD Equity Act provides a structured blueprint for governance, its implementation faces significant practical challenges. Establishing a federally coordinated, interoperable data infrastructure requires substantial initial funding and the reconciliation of fragmented state-level privacy regulations. Furthermore, the viability of mandatory audits depends on developing standardized metrics for fairness that can be applied across diverse clinical settings. Acknowledging these hurdles, including the technical difficulty of data integration and the need for sustained political will, is essential to transitioning this framework from a conceptual model into a functional policy reality.

Global implications

Ethical governance of AI in CVD has global relevance. International collaboration, supported by privacy‑preserving and federated learning approaches, can enhance algorithm performance across diverse populations while respecting data sovereignty [14]. Alignment with global digital health strategies facilitates shared learning and reduces the risk of widening global cardiovascular inequities [13].

Conclusion

AI holds substantial promise for transforming CVD prevention, but its benefits depend on robust ethical governance and equity‑centered policy. Current US regulatory approaches remain insufficient to ensure fair and transparent AI deployment at scale. The proposed AI‑CVD Equity Act offers a policy pathway to integrate AI into CVD prevention while safeguarding equity, accountability, and public trust. By synchronizing these national efforts with international digital health initiatives and privacy-focused data methods, this framework ensures that AI deployment advances, rather than undermines, population cardiovascular health on a global scale. 

## References

[1] (2026). World Health Organization. Cardiovascular Diseases (CVDs). https://www.who.int/news-room/fact-sheets/detail/cardiovascular-diseases-(cvds).

[2] (2026). Centers for Disease Control and Prevention. Fast Facts: Health and Economic Costs of Chronic Conditions. Chronic Disease.

[3] Rajkomar A, Dean J, Kohane I (2019). Machine learning in medicine. N Engl J Med.

[4] Topol EJ (2019). High-performance medicine: The convergence of human and artificial intelligence. Nat Med.

[5] Khera R, Oikonomou EK, Nadkarni GN, Morley JR, Wiens J, Butte AJ, Topol EJ (2024). Transforming cardiovascular care with artificial intelligence: From discovery to practice: JACC state-of-the-art review. J Am Coll Cardiol.

[6] Attia ZI, Friedman PA, Noseworthy PA (2019). Age and sex estimation using artificial intelligence from standard 12-lead ECGs. Circ Arrhythm Electrophysiol.

[7] Johnson KW, Torres Soto J, Glicksberg BS (2018). Artificial intelligence in cardiology. J Am Coll Cardiol.

[8] Obermeyer Z, Powers B, Vogeli C, Mullainathan S (2019). Dissecting racial bias in an algorithm used to manage the health of populations. Science.

[9] (2025). U.S. Food and Drug Administration. AIML-SaMD-Action-Plan.pdf. https://eu.docworkspace.com/d/sIEa234bMAem-1McG.

[10] Owusu-Mensah P, Richwine C (2012). Electronic public health reporting among non-federal acute care hospitals. ASTP Health IT Data Brief [Internet].

[11] Benjamens S, Dhunnoo P, Meskó B (2020). The state of artificial intelligence-based FDA-approved medical devices and algorithms: An online database. NPJ Digit Med.

[12] Char DS, Shah NH, Magnus D (2018). Implementing machine learning in health care - Addressing ethical challenges. N Engl J Med.

[13] (2026). WHO. Ethics and Governance of Artificial Intelligence for Health. https://www.who.int/publications/i/item/9789240029200 .

[14] (2026). WHO. Global Strategy on Digital Health 2020-2025. https://www.who.int/docs/default-source/documents/gs4dhdaa2a9f352b0445bafbc79ca799dce4d.pdf.

